# Supporting smoking cessation in chronic obstructive pulmonary disease with behavioral intervention: a randomized controlled trial

**DOI:** 10.1186/1471-2296-14-91

**Published:** 2013-06-27

**Authors:** Peian Lou, Yanan Zhu, Peipei Chen, Pan Zhang, Jiaxi Yu, Ning Zhang, Na Chen, Lei Zhang, Hongmin Wu, Jing Zhao

**Affiliations:** 1 The Xuzhou Center for Disease Control and Prevention, 142 West Erhuan Road, Xuzhou City, Jiangsu Province, People's Republic of China 221006; 2 Department of Respiratory Medicine, Hospital of Xuzhou medical college, 99 West Huaiai Road, Xuzhou City, Jiangsu Province, People's Republic of China 221006

**Keywords:** Chronic obstructive pulmonary disease, Smoker, Behavioral intervention, Smoking cessation, Risk factors

## Abstract

**Background:**

Cigarette smoking is the major risk factor for chronic obstructive pulmonary disease (COPD). But a fewer smoking cessation measures were conducted in communities for smokers with COPD in China. The aim of our study was to assess the preventive effects of behavioral interventions for smoking cessation and potential impact factors in smokers with COPD in China.

**Methods:**

In a randomised controlled smoking cessation trial 3562 patients with COPD who were current smoker were allocated to intervention group received behavioral intervention and control group received the usual care for two years. The primary efficacy endpoint was the complete and continuous abstinence from smoking from the beginning of month 24 to the end of month 30. Participants were followed up at month 48.

**Results:**

Continuous smoking abstinence rates from month 24 to 30 were significantly higher in participants receiving behavioral intervention than in those receiving usual care (46.4% *vs* 3.4%, p < 0.001). Continuous abstinence rates from months 24 to 36 (45.8% *vs* 4.0%) and months 24 to 48 (44.3% *vs* 5.1%) were also higher in participants receiving behavioral intervention than in those control group. Family members or family physicians/nurses smoking were first identified to influence smoking cessation.

**Conclusions:**

Behavioral intervention doubled the smoking cessation rate in patients with COPD and was complied well by the general practitioners. The family members and family physicians/nurses smoking were the main risk factors for smoking cessation.

**Trial registration:**

Chinese Clinical Trials Registration (ChiCTR-TRC-12001958).

## Background

Chronic obstructive pulmonary disease (COPD) is a progressive systemic inflammatory disease that is usually an abnormal response to noxious particles and gases (more often, tobacco smoke) in susceptible individuals. Cigarette smoking is a worldwide risk factor for COPD [1,2], which accounts for 80-90% of COPD patients [3]. Løkke and colleagues found that smoking significantly increased the cumulative incidence of COPD in a 25 year follow up study [4]. The highest incidence for all stages of COPD was 35.5% that occurred in continuous smokers, while the incidence of never smokers was only 7.8% [4]**.** Comparing cigarette smoking status in COPD patients, Zhou et al. found that the greater amount of smoking, the deeper inhalation into the airway and start smoking at earlier age had the greater risks of COPD [5]. Kanner et al. investigated mild stage of COPD patients continuously smoking, the speed of forced expiratory volume in 1 second (FEV_1_) was declined when suffered from the lower airflow illness. Stopping smoking protected these people with mild COPD from this additional loss of lung function [6].

Quitting smoking is the most cost-effective method to prevent lung function deterioration for COPD patients. A longitudinal cohort study showed that continuous smokers had a much steep decline of lung function than those stopped smoking, while never smokers had the best lung function [7]. Lung Health Study confirmed that smoking cessation could reduce smoking-related decline in lung function [8,9]. When COPD patients with severely impaired lung function stopped smoking, their lung function might be not recovered, but the subsequent decline tend to be normal [10,11]. On the other hand, smoking cessation also improved airway hyperresponsiveness for COPD patients [12]. Smoking cessation at the early stage was able to benefit COPD prognosis [7,8,11,13], which was more effective than stop smoking at the later stages[14]. These data suggested the importance of COPD patients quit smoke as early as possible [5].

In China, over 40 million people suffered from COPD, and more than 1.28 million died from it every year [15]. About 80-90% of patients with COPD were smokers [16]. Although efficacious smoking cessation methods have been established for patients with COPD [17-19], no more stringent advice or pharmacological therapies have been applied for COPD patients to quit smoking compared with general smokers [20], and examined the influencing factors of community-based smoking cessation trial in current smokers with COPD in China especially.

In present study, we conducted a randomized controlled trial to assess the efficacy of a two-year course of behavioral interventions on helping patients with COPD to quit smoking, and explore potential factors potentially barring smoking cessation.

## Methods

### Study design

**Figure 1:**
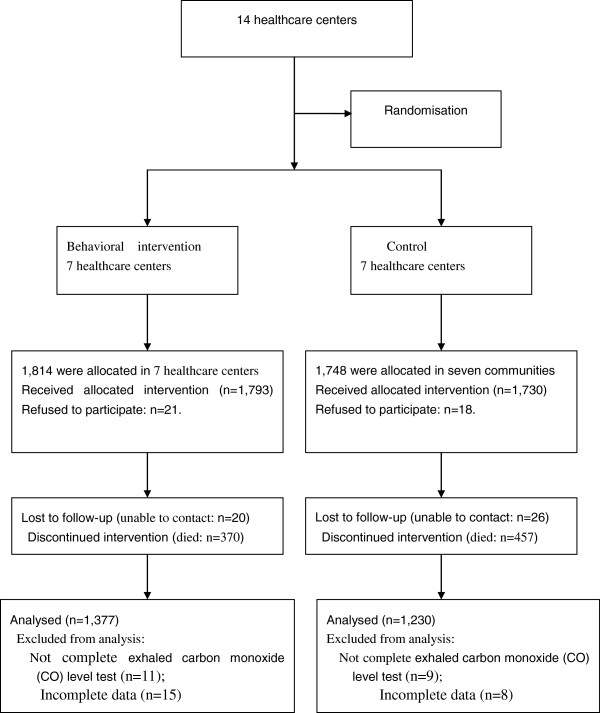
Consort figure of the trial profile.

The study was a randomized controlled trial conducted form from January 2008 to May 2012, which involves three months patients’ recruitment, two years’ intervention, two years’ monitoring. Recruitment of practices took place in 28 communities based on our previous epidemiological study [21]. Fourteen healthcare centers enrolled in the study; General healthcare centers in the intervention group received support to implement the behavioral intervention program, whereas the control healthcare centers delivered usual care. Randomization took place on healthcare center level. The healthcare centers were classified in two classes: with high or low task delegation from general practitioners to nurses. The healthcare centers in the classes were then randomly allocated to the groups (See: consort Figure 1).

A two-sided P value <0.05 was used. Based on a two-sided Type 1 error (α) = 0.05, with an 80% power to detect a 25% relative reduction in quitting rates, allowing 20% loss during follow-up, we need that each group should contain a minimum of 7 healthcare centers s and at least 50 patients with COPD per group.

This study was approved by the Ethics Committee of the Xuzhou Center for Disease Control and Prevention and the Regional Ethical Vetting Board, Xuzhou, China. In addition, agreement was received from all of the relevant health centers. Informed consent was obtained from all participants.

### Subjects

Patients were recruited by their family physicians from 14 healthcare units in rural area of Xuzhou city, China, from January to March 2008. Patients had to meet the following criteria at baseline: COPD diagnosed by the standards set forth by the Global Initiative for Chronic Obstructive Lung Disease (GOLD) [22]. Which means all participants underwent standard measurements of lung function (post-bronchodilator forced expiratory volume in 1 s (FEV_1_)/forced vital capacity (FVC) <70%) by the County People’s Hospital. Reversibility of airways obstruction was measured according to standard America Thoracic Society guidelines (over 15% and at least 400 ml improvement in FEV_1_ after 400 μg salbutamol via a spacer) [23]. The study population consisted of current smokers with all stage COPD. All patients were aged 35 years or older, had smoked 1 cigarette or more per day for the previous year, and had not stopped smoking for more than 3 months during that year. We excluded participants who had any serious or unstable medical disorders such as psychiatric that might affect lung function or they had a current diagnosis of major depression.

Participants were considered as loss to follow-up if they could not contact, died, move to another place, withdraw consent, refuse to proceed, invalid data, or unable to complete the study due to other reasons.

### Behavioral interventions

During the screening phase, individuals selected a date to stop smoking (target day) and were told not to attempt to stop before this day. Patients were also told the aim of the study.136 general practitioners, working at 7 different healthcare centers of the intervention group, followed 6 hours training in behavioral interventions for quitting smoking. The training included: ingredients of tobacco, the potential harmful of smoking, smoking and COPD, harmful of exposure to tobacco smoke, smoking's impact on the economy, weigh the pros and cons of smoking and smoking cessation, why the COPD patients need quit smoke, benefits of quitting smoking, how to deal with smoking cravings, how to preventing relapse smoking, how to develop smoking cessation programs, how to help them quit smoking and the motivational interview.

The teams of general practitioners (including assistants and nurses) were established in the healthcare centers of intervention group. They were responsible for supervising and advising patients with COPD registering in their healthcare centers to quit smoke. The tasks of them included home visiting patients with COPD at least once a week, to obtain the current condition of quitting smoking, tell the patients how to do next, and record the time of quitting smoking. If a patient has quitted smoking, the general practitioners were to follow-up the patient once a week at the first month and afterwards once a month until the end the study. Meanwhile, they also were responsible for examining the exhaled carbon monoxide (CO) level for COPD patients. Every month, the participants were asked to meet together once to discuss relevant questions for quitting smoking and share the experience of quitting smoking. The professional group (including respiratory, rehabilitation, nutrition, sports and psychology specialists) routinely visited the COPD patients in healthcare centers every two months. The general practitioners in the healthcare centers reported the follow-up status of patient with COPD and smoking cessation conditions once a month. The professional group assessed smoking cessation conditions, developed the focus of the next follow-up period for each patient, and sent the focus to the teams of the general practitioners. The focus was how to improve patients’ quitting smoking during next follow-up period. The professional group also provided other physiological supporting, including the benefits of smoking cessation and how to deal with obesity. This study was lasted four years. During the follow-up period, personnel support was offered with easily access for all participants. All participants were tested for exhaled carbon monoxide (CO) level at the baseline and every six months in the follow-up period.

At the baseline, all patients were interviewed by the general practitioners and the professional group at healthcare centers. All participants were asked if they were willing to quit smoking. The participants who would like to quit smoking were given a booklet that included:

– how many harmful ingredients are in cigarettes;

– smoking and disease (included: COPD);

– Benefits of quitting smoking;

– how to deal with cravings for smoking;

– Ways to quit smoking.

For the patients in intervention group, we strongly encouraged them to stop smoking, and received brief smoking cessation advice after the baseline interview, which consist of 5–8 minutes’ discussion with member of professional group about their smoking habits. Smoking cessation advice was focused on the risk of developing COPD, lung cancer, coronary artery disease, and the harmful effects to other family members. If the participants wanted to stop smoking, we provided them a plan to quit smoking. For example, we asked the participants to set date to stop smoking (target day). If the patients currently smoked twenty cigarettes per day, they could decrease two cigarettes per day or two days. If the patients did not want to stop smoking, we encouraged them to stop smoking within six months. They could decrease one cigarette per day or one week. It was suggested for all participants to postpone the time as long as possible when they want to smoke. Drinking water, talking with someone, or turning their attention on other things were recommended as effective ways to postpone smoking time. No pharmacological treatment for smoking addiction was provided in the current study.

### Smoking status

An exhaled carbon monoxide (CO) measurement was taken at baseline and every six month in the follow-up period to confirm smoking status at healthcare centers. The primary efficacy measure was continuous abstinence from smoking for 2 years from the start of month 24 to the end of month30. We defined continuous abstinence as a participant report of zero cigarettes per day for at least 6 months and confirmed by exhaled carbon monoxide values of 10 parts per million

Secondary measures of efficacy were continuous abstinence during month 24–36 and 24–48. Continuous abstinence for months 24–48 was defined by participants being continuously abstinent during months 24–36, having a diary cigarette count of zero during weeks 37–48, and having exhaled carbon monoxide values of 10 ppm or less at month 48.

### Assessment of influencing factors

All participates were asked to complete a questionnaires that included age, sex, current employment status, education level, marital status, physical activity, alcohol use, number of smoking family member, number of cigarettes smoked, motivation to stop smoking, smoking pack years (average daily numbers of cigarettes smoked divided by 20 and multiplied by the smoking years). Comorbidities including chronic bronchitis or emphysema, asthma, other lung disease, diabetes, treatment for blood pressure, stroke, coronary heart disease (angina or heart attack), or other heart disease and conditions were recorded on patient’s baseline reports. Education was categorized into below high school, high school, or above high school levels. Alcohol drinking was defined as the consumption of at least 30 g of alcohol per week for one or more years. The height and weight were measured, and body mass index (BMI) was calculated (BMI = weight in kilograms divided by height in meters squared). BMI was categorized as underweight (≤18.5 kg/m^2^), normal (18.5 to ≤24.0 kg/m^2^) or overweight/obese (≥24.0 kg/m^2^) [24]. Mental health (depression and anxiety) was evaluated by the Hospital Anxiety and Depression Scale (HADS) [25]. Dyspnea was measured using the Medical Research Council (MRC) dyspnea scale [26] and FagerstrÖm Test for Nicotine Dependence (FTND) [27].

### Usual care

Participants in control group were treated by healthcare providers or general practitioners as usual manner. The content and number of usual care services were not standardized. Participants were follow-upped once every 2 months, and asked whether the symptoms aggravated, what medication they used, etc.

### Statistical analyses

We did all analyses on the intention-to-treat population, which consisted of patients who took at least one month of our study. All participants who withdrew from the study were taken to be smokers thereafter.

SPSS for Windows version 11.5 (SPSS Inc.) was used for data analysis. Tests were considered as significant when *P* < 0.05. Baseline demographic and smoking characteristics as well as mean scores of MRC, and mental health were compared to assess potential differences between intervention group and control. T-test was used to analyze the differences in mean scores between the groups. A chi-squared test was used to evaluate the statistical significance of smoking cessation rates between the two groups. We studied the effect of age, sex, education level, household income, alcohol user, depression, anxiety, smoking pack-years, Nicotine addiction, dyspnea, smoking history of family members and smoking history of the responsible doctor or nurse of patients with multivariate logistic regression. These variables were added to the basic model including intervention group assignment and centre. Candidate variables (*P* < 0.05) were entered into the multiple logistic regression models analysis to analyze risk factors affecting quit smoking. Odds ratios (OR), confidence intervals (CI) and p values were calculated.

## Results

### General characteristics of the participants at the baseline

The two groups were drawn from a single district and were similar in annual averaged income, access to health services and main demography at baseline (Table 1).

**Table 1:** Baseline characteristics within healthcare units

	**Intervention group**	**Control group**
No of units	7	7
Population	301,785	298,467
Annual per capita income (Yuan)	21,345 ± 464	21,545 ± 471
Per capita housing area (m^2^)	25.6 ± 6.4	25.6 ± 6.5
medical staff	2,162	2,147
Medical staff constitute		
Doctor	654 (38.1)	648 (37.0)
Nurse	1,041 (38.7)	1,058 (41.0)
Medical staff educational background		
Undergraduate or above	1,576	1,573
Junior college	482	479
Medical staff professional titles		
Senior title	559	855
Intermediate title	881	878

Figures are numbers (percentages) unless stated otherwise**.**

Among 3562 smokers, 26.8% did not complete the follow-up visit, i.e. 46 subjects had moved away from the region, 39 declined to participate in the follow-up visit for miscellaneous reasons, 20 did not complete exhaled carbon monoxide (CO) level test, 23 were incomplete data, and 827 died (intervention: 370 *vs* control: 457; χ^2^ = 28.76, P < 0.001). The total number of lost in behavioral intervention and control group were 437 and 518, respectively. Significant difference in lost rates was observed between behavioral intervention and control group (χ^2^ = 13.94, P < 0.001). There were no statistically significant differences in the smoking history, age and sex between those who attended the follow up and non-responders. Finally, 2607subjects were analyzed in the study, 1377 in behavioral intervention group and 1230 in control group (Figure 1). In behavioral intervention group, the mean ages, median smoking years, median nicotine dependence scales and median MRC scales were 61.6 ±10.2 years, 37.8 ±11.7pack-years, 5.1 ±2.1 and 2.4 ±1.5, respectively. In the control group, the mean age of 61.5 ± 10.1 years, median smoking years of 37.6 ±11.5 pack-years, and median nicotine dependence scales of 5.0 ±1.9. No statistically significant differences in these indicators were observed between the two groups. Other descriptive characteristics for behavioral intervention and control group were shown in Table 2. Which showed no statistically significant differences between the intervention and control groups (all Ps > 0.05).

**Table 2:** The characteristics and smoking rates at the baseline

**Variables**	**Intervention group (n = 1,814)**	**Control group (n = 1,748)**
Sex		
Men	868 (100)	840 (100)
Women	946 (100)	908 (100)
Age		
40~	292 (100)	279 (100)
50~	370 (100)	358 (100)
60~	507 (100)	490 (100)
70~	478 (100)	463 (100)
80~	167 (100)	158 (100)
Education level		
High	159 (100)	152(100)
Middle	327 (100)	323 (100)
Low	1328 (100)	1273 (100)
Household income		
High	148 (100)	146 (100)
Middle	1529 (100)	1466 (100)
Low	137 (100)	136 (100)
Alcohol user		
Yes	659 (100)	628 (100)
No	1155(100)	1120 (100)
Comorbidities		
Yes	473 (100)	459 (100)
No	1341 (100)	1289 (100)
Depression		
Yes	640(100)	622(100)
No	1174(100)	1126(100)
Anxiety		
Yes	343 (100)	337 (100)
No	1471 (100)	1411 (100)
Pack years		
<30	604 (100)	572 (100)
30~	648 (100)	631 (100)
40~	562 (100)	545 (100)
Nicotine addiction (FagerstrÖm score)		
0~3	526 (100)	502 (100)
4~6	648 (100)	654 (100)
≥7	640 (100)	592 (100)
MRC scales		
0	91 (100)	89 (100)
1	358 (100)	346 (100)
2	524 (100)	499 (100)
3	502 (100)	486 (100)
4	339 (100)	328 (100)
Smoking history of family members		
Yes	1128 (100)	1075 (100)
No	686 (100)	673 (100)
Smoking history of the responsible doctor or nurse		
Yes	798 (100)	778(100)
No	1016 (100)	970 (100)

*MRC* Medical Research Council.

### Quit smoking rates

The rates of continuous abstinence from month 24 to the end of month 30 was higher in the intervention group than that in the control group ( 46.4% vs 3.4% p<0.001). 639 out of 1377 (46.4% ) participants receiving behavioral intervention remained abstinent compared with 42 out of 1230 (3.4%) receiving usual care. Rates of continuous abstinence were significantly higher with behavioral intervention than with usual care throughout the 24-month intervention phase and at the 48 month follow-up visit (Table 3). At the 48 month follow-up, more participants receiving behavioral intervention remained abstinent than those receiving usual care (44.3% vs 5.1% , p<0.001). Behavioral intervention was significantly better than usual care at months 36–48 during the follow-up phase (p<0.001).

**Table 3:** Abstinence rates during intervention and follow-up phases

**Month**	**Continuous abstinence**		**P**
	**Intervention group**	**Control group**	
	**(n = 1377)**	**(n = 1230)**	
6	79(5.7%)	3(0.2%)	<0.001
12	198(14.4%)	11(0.9%)	<0.001
18	342(24.8%)	24(2.0%)	<0.001
24	508(36.9%)	36(2.9%)	<0.001
30	639(46.4%)	42(3.4%)	<0.001
36	630(45.8%)	49(4.0%)	<0.001
42	622(45.2%)	57(4.6%)	<0.001
48	610(44.3%)	63(5.1%)	<0.001

No cigarettes from month 6 onwards.

The significant differences of continuous smoking abstinent rates were consistently observed for all subgroups (such as different ages, gender, education levels, household income, alcohol user and comorbidities etc.), i.e. higher smoking cessation rate in intervention group than that in control group (Table 4).

**Table 4:** Observed frequencies of abstinence from month 31 to end of month 48

**Variables**	**Smoking cessation group (n= 1377)**	**Control group ** **(n= 1230)**	**Difference (95% CI)**	**P value**
Sex				
Men (%)	265/659(40.2)	28/601 (4.7)	35.5(23.6-49.8)	P<0.001
Women	345/718 (48.0)	35/628 (5.6)	42.4(27.8-58.6)	P<0.001
Age				
40~	87/221( 39.4)	5/197 (2.5)	36.9(29.5-45.6)	P<0.001
50~	118/280 (42.1)	11/250 (4.4)	37.7(26.5-50.1)	P<0.001
60~	171/385 (44.4)	17/343 (5.0)	39.4(26.6-53.4)	P<0.001
70~	167/362 (53.8)	17/323 (5.3)	48.5(37.8-60.7)	P<0.001
80~	67/129 (51.9)	13/117 (11.1)	40.8(33.5-49.2)	P<0.001
Education level				
High	74/143 (51.7)	8/129 (6.2)	45.5(37.7-54.3)	P<0.001
Middle	137/313 (43.8)	14/280 (5.1)	38.7(25.4-53.2)	P<0.001
Low	399/921 (43.3)	41/821 (5.0)	38.3(20.6-57.3)	P<0.001
Household income				
High	48/112 (42.8)	7/114(6.1)	36.7(30.3-43.9)	P<0.001
Middle	524/1173 (44.7)	53/1051 (5.0)	39.7(20.5-59.8)	P<0.001
Low	38/92 (41.2)	3/65 (4.6)	36.6(31.3-42.4)	P<0.001
Alcohol user				
Yes	201/498 (40.4)	17/434 (3.9)	36.5(25.6-49.4)	P<0.001
No	409/879 (46.5)	46/796 (5.8)	40.7(26.4-56.1)	P<0.001
Comorbidities				P<0.001
Yes	68/136 (50.0)	15/121 (12.4)	37.6(25.3-55.4)	P<0.001
No	542/1241 (43.4)	48/1109 (3.9)	39.5(20.7-58.6)	P<0.001
Depression				
Yes	95/513/(18.5)	13/518(2.5)	16.0(12.7-20.5)	P<0.001
No	515/864(59.6)	50/712(7.0)	52.6(41.9-63.5)	P<0.001
Anxiety				P<0.001
Yes	81/258 (31.4)	9/230 (4.0)	27.4(20.1-35.7)	P<0.001
No	529/1119 (47.3)	54/1000 (5.4)	42.1(33.3-51.1)	P<0.001
Pack years				
<30	266/564 (47.2)	32/504 (6.3)	40.9(32.4-50.4)	P<0.001
30~	200/454 (44.1)	18/406 (4.4)	39.7(31.8-48.2)	P<0.001
40~	144/359 (40.1)	13/320 (4.1)	36.0(29.7-43.3)	P<0.001
Nicotine addiction (FagerstrÖm score)				
0~3	243/432(56.3)	25/386 (6.5)	49.8(35.6-64.6)	P<0.001
4~6	233/486(47.9)	23/434 (5.3)	42.6(29.1-56.7)	P<0.001
≥7	134/459 (29.2)	15/320 (4.7)	24.5(13.4-36.6)	P<0.001
MRC scales				
0	**21 /70(30.0)**	**2/71 (2.8)**	27.2(22.6-32.3)	P<0.001
1	107/272 (39.3)	7/243(2.9)	36.4(27.5-46.3)	P<0.001
2	175 /398(44.0)	17/356 (4.8)	39.2(27.7-51.7)	P<0.001
3	178/380 (46.8)	20/339 (5.9)	40.9(28.1-54.4)	P<0.001
4	129/257 (50.2)	15/221 (6.8)	43.4(31.1-56.7)	P<0.001
Smoking history of family members				
Yes	247/816 (30.3)	28/729(3.8)	26.5(13.2-40.1)	P<0.001
No	363/561 (64.7)	37/501 (7.4)	57.3(38.9-76.2)	P<0.001
Smoking history of the responsible doctor or nurse				
Yes	214/589 (36.3)	22/526 (4.2)	32.1(21.7-43.9)	P<0.001
No	396/788 (50.3)	41/704 (5.8)	44.5(32.1-57.6)	P<0.001

### Verified quit smoking

The successful smoking cessation during the 4-year follow up was confirmed by a low exhaled CO level for 610 subjects. The mean exhaled CO in sustained quitters was significantly lower (mean = 3.7 ppm, SD = 1.5) compared with continuous smokers (mean = 15.7 ppm, SD = 5.1, *p*<0.001). Subjects who were continuous smokers also reported a reduced cigarettes smoked per day at the end of 4-year follow up (mean = 14.5, SD = 7.2) compared to the baseline (mean = 20.5, SD = 9.2).

### Risk factors affecting smoking cessation

Independent predictors of successful smoking cessation included women, older age, no-drinkers, comorbidities, lower HADS-anxiety (HADS-A), lower HADS -depression (HADS-–D), less smoking pack years, lower FagerstrÖm score, and higher MRC score (Table 5). Patients were more likely to quit smoking if they had the family physicians/nurses nonsmoking or no family member smoking (Table 5). In other words, the family physicians/nurses and family members smoking were two strong predictors of failure to quit smoking. Other confounders might also play a role. The multiple logistic regression analysis showed significant effects for alcohol drinkers, comorbidities, HADS-A, HADS-D, smoking pack years, FagerstrÖm score, MRC score, family members smoking, and the family physicians/nurses smoking as predictors of smoking cessation by adjusting age and gender (Table 5). The family members smoking was the strongest predictor of sustained smoking (OR = 12.1).

**Table 5:** Multiple logistic regression analyses for factors associated with failure smoking cessation

**Variable**	**OR**	**95% CI**	**P values**
Gender			
Female	1.00		<0.001
Male	1.50	1.28-1.77	
Comorbidities			
No	1.00		0.000
Yes	1. 19	1.08-1.38	
Alcohol drinking			
No	1.00		<0.001
Yes	0.61	0.51-0.72	
Depression			
No	1.00		0.003
Yes	1.26	1.09-1.40	
Anxiety			
No	1.00		0.000
Yes	1.25	1.07-1.43	
Pack years			
<30	1.00		
30~	1.08	1.01-1.24	0.009
40~	1.55	1.34-2.21	0.000
Nicotine addiction (FagerstrÖm score)			
0~3	1.00		
4~6	1.25	1.09-1.49	0.002
≥7	1.62	1.29-2.15	0.000
MRC scales			
4	1.00		
3	1.37	1.12-1.66	0.001
2	1.52	1.28-1.81	0.000
1	1.65	1.35-2.01	0.000
0	1.72	1.37-2.21	0.000
Family member smoking			
No	1.00		0.000
Yes	12.1	18.7-35.2	
Family doctor smoking			
No	1.00		0.000
Yes	4.7	2.26-10.4	

*MRC* Medical Research Council.

## Discussion

Our results show that the behavioral intervention group had the high rate of abstinence from smoking over 24 months in smokers with COPD compared with the control group. This advantage over usual care continued for 24 months after discontinuation of the behavioral intervention. It also shows that the family physicians/nurses and family members smoking were two strong predictors for failure to quit smoking.

Overall, the population-attributable risk of COPD related to cigarette smoking was higher (about 45%)[28,29]. Smoking cessation is an efficient way of slowing down COPD progression. The Lung Health Study (LHS) examined the effects of tobacco intervention on COPD progression among current smokers [8]. The smokers with mild to moderate COPD were followed up at 4-month intervals to check on smoking status and the compliance with smoking cessation determined by self-report and verified by measuring expired carbon monoxide and salivary cotinine levels. Over the 4 years of follow-up, 22% of patients with COPD were continuous abstinence smokers in two groups receiving special tobacco treatment. In contrast, only 5% of the patients in usual care group became continuous abstinence smokers. Smoking habits by original Lung Health Study treatment groups tended to converge, but 93% of participants who were abstinent throughout the Lung Health Study were still abstinent at 11 years [30]. Our behavioral intervention showed a consistent and significantly higher continuous smoking abstinence rate (44.3%) in patients with COPD compared with control group (5.7%) after four years. We also found that 22.6% of smokers reduced the amount of cigarettes smoked. These data highlighted the feasibility and efficacy to conduct community-based smoking cessation in patients with COPD. The long term follow-up studies are warrant to evaluate the longitudinal effects of smoking cessation.

Our study also identified several important factors which potentially impact smoking cessation, including women, older, non-drinkers, comorbidities and dyspnea. Patients with depression, anxiety, higher smoking pack years and nicotine addiction were difficult to quit smoking. These results provided additional information and potential methods to improve the management of COPD patients. For example, a previous study found that female smokers with COPD might experience a faster decline in lung function than male smokers [31]. Another study indicated that female smokers had larger gains in lung function when stopped smoking compared with male quitters [32]. These data strongly suggested that smoking cessation was particularly important for women because they had increased susceptibility to COPD progression if continued smoking, but may obtain more benefits if quitting smoking compared with male smokers. These results could be used as strong biological evidence and tools in conducting community-based intervention to convince women quit smoking. It was also consistent with our observational study that women with COPD were relatively easy to quit smoking.

For the first time, we found two important factors (family members smoking and family physicians smoking) were significantly associated with smoking cessation in China. Luker conducted a systematic review to assess the effectiveness of family-focused smoking cessation interventions for patients with COPD [33]. However, no conclusion was obtained about the effectiveness of a family-focused intervention, including factors of marital status, smoking status of household members and support for smoking cessation. The negative impact of smoked doctors or nurses on smoking cessation of COPD patients displayed a great challenge we faced on community-based intervention. The doctors or nurses usually provided a positive attitude and health knowledge to persuade COPD patients to quit smoking, which played a critical role in improving the efficacy of smoking cessation intervention. The message that “quitting smoking benefits your health or disease progression” may not be convincible if provided by doctors or nurses who are smokers. On the other hand, our data also suggested that there is a lot of space to improve the smoking cessation if we can firstly persuade the doctor or nurse to quit smoking.

The strengths of current study included a community-based intervention design, large sample size, randomly selected intervention and control groups, and low rate of loss of follow-up. The accurate smoking cessation data obtained by both questionnaires and exhaled CO measurement also made the current results more reliable. One limitation was a moderate duration (four years) that could not evaluate the long-term efficacy of smoking cessation. No detailed data on comparison lung functions and other clinical outcomes of COPD patients before and after smoking cessation is also limited, which may potentially reduce the efficacy of quitting smoking. Previous studies have demonstrated that smoking cessation was associated with a slower decline in lung function and reduced risk of hospitalization and total mortality in another study [8]. Knowing smokers’ lung function being declining might help motivate patients to quit smoking [18,34,35]. This data strongly encouraged us to adopt lung function detection in the community-based intervention to improve the efficacy of smoking cessation.

## Conclusions

In summary, our community-based study revealed two-year behavior intervention significantly decreased smoking rate in currently smoked patients with COPD. Several important factors have been identified to positively or negatively impact smoking cessation, especially the smoking behaviors of other family members and the responsible doctors or nurses that were first associated with smoking cessation. Long-term follow-up for smoked patients with COPD and validating their lung functions may further improve the effect of community-based intervention, and benefit the prognosis of COPD patients.

## Competing interests

The authors declare that they have no competing interests.

## Authors’ contributions

LZ and PL participated in writing the title and abstract, contributing to the writing of the manuscript drafts and reviewing the full text. YN conceptualized the study, participated in its design, title and abstract screening, full text screening, data extraction and analysis, and drafting of the manuscript. PC and PZ performed literature searches, participated in title and abstract screening, full text review, and contributed to the manuscript drafts. JY and NZ conceptualized the study; participated in its design, title and abstract screening, full text screening; and contributed to the manuscript drafts. NC and LZ contributed to the conception of the study, participated in the study design, and contributed to the manuscript drafts. HW and JZ were the lead authors of the original review, contributed to the conception of the study, participated in the study design, and contributed to the manuscript drafts. All authors have read and approved the final manuscript.

## Pre-publication history

The pre-publication history for this paper can be accessed here:

http://www.biomedcentral.com/1471-2296/14/91/prepub
